# Modulation of Obesity and Insulin Resistance by the Redox Enzyme and Adaptor Protein p66^Shc^

**DOI:** 10.3390/ijms20040985

**Published:** 2019-02-24

**Authors:** Stefano Ciciliot, Gian Paolo Fadini

**Affiliations:** 1 Veneto Institute of Molecular Medicine, 35128 Padova, Italy; stefano.ciciliot@gmail.com; 2 Department of Medicine, University of Padova, 35128 Padova, Italy

**Keywords:** diabetes, metabolic syndrome, adipose tissue, muscle, glucose tolerance, aging, oxidative stress

## Abstract

Initially reported as a longevity-related protein, the 66 kDa isoform of the mammalian *Shc1* locus has been implicated in several metabolic pathways, being able to act both as an adaptor protein and as a redox enzyme capable of generating reactive oxygen species (ROS) when it localizes to the mitochondrion. Ablation of p66^Shc^ has been shown to be protective against obesity and the insurgence of insulin resistance, but not all the studies available in the literature agree on these points. This review will focus in particular on the role of p66^Shc^ in the modulation of glucose homeostasis, obesity, body temperature, and respiration/energy expenditure. In view of the obesity and diabetes epidemic, p66^Shc^ may represent a promising therapeutic target with enormous implications for human health.

## 1. Introduction

The p66^Shc^ protein is encoded by the *Shc1* locus, together with two shorter isoforms known as p52^Shc^ and p46^Shc^ [1,2]. While the last two proteins are generated by the same mRNA using different translation initiation sites [2], p66^Shc^ is produced from a different exon arrangement at the 5′ end. The structure of p66^Shc^ and of the *Shc1* locus were extensively reviewed by different authors and will not be discussed in detail in this review [3,4,5]. The three Shc isoform proteins share a common structure, which comprises a phosphotyrosine-binding domain (PTB), a collagen homology 1 (CH1) domain rich in prolines, and a sarcoma homologous type 2 domain (SH2). Shc protein family members are present in mammals, amphibians, fishes, insects (*D. Melanogaster*), nematodes (*C. Elegans*), and yeasts, and a typical characteristic of them is to have PTB and SH2 domains in the same order from the N- to C-terminals [3,5]. From an evolutionary point of view, p66^Shc^ appears to be the most recent isoform, as it is found in vertebrates, but not in yeasts, nematodes, and insects. Unlike p52^Shc^ and p46^Shc^, p66^Shc^ has an additional collagen homology region (CH2); moreover, p46^Shc^ does not have a cytochrome c binding domain (CB), which is shared between p52^Shc^ and p66^Shc^.

### 1.1. Role of p66^Shc^ in Signal Transduction

**Figure 1:**
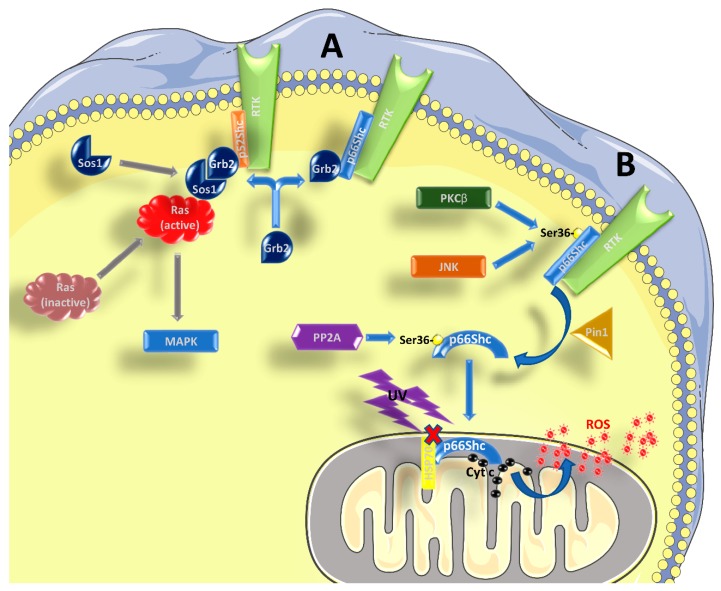
Role of p66^Shc^ in signal transduction. (**A**) p52^Shc^ and p46^Shc^ are activated by phosphorylation in tyrosine residues within their CH1 domain, when bound to RTKs and possibly other receptors. Subsequently, the recruitment of the Grb2/Sos1 complex allows for the activation of Ras and the MAPK pathway. p66^Shc^ can compete with the other two isoforms for the binding of Grb2, interfering with Ras activation. (**B**) After being activated by RTKs, and the concomitant phosphorylation in Ser^36^ by kinases such as PKCβ or JNK, p66^Shc^ is subjected to cis-trans isomerization by Pin1. It then translocates to the inter-membrane space of the mitochondrion, after being dephosphorylated by PP2A. Without stimulation, p66^Shc^ is bound to other proteins, like HSP70, and therefore is inactive. After stimulation with UV-light or H_2_O_2_, p66^Shc^ can bind to cytochrome c and contribute to the formation of ROS. See the main text for further details.

The three aforementioned Shc proteins are also different in terms of the signaling pathway wherein they are involved. It is well known that p52^Shc^ and p46^Shc^ are able to transduce the signal from tyrosine-kinase receptors (RTKs) to Ras and mitogen-activated protein kinase (MAPK) pathways [1,6] (Figure 1A). Shc binding to RTKs causes a phosphorylation of three tyrosine residues in their CH1 domain, which is required for the recruitment of the Grb2/Sos1 complex (growth factor receptor-bound protein 2 and son of sevenless 1) at the SH2 domain, which in turn leads to the activation of Ras [4], as Sos1 is a guanine nucleotide exchange factor (GEF). Given its structure, p66^Shc^ should be able to form the same complexes and activate Ras. However, many studies have indicated that p66^Shc^ has an inhibitory role on the Ras-MAPK pathway, regardless its ability to bind Grb2 [2,6,7,8,9]. It has been proposed that p66^Shc^ competes with p52^Shc^ and p46^Shc^ for the binding with Grb2, causing the disruption of the Grb2/Sos1 complex, and in this context it seems that the phosphorylation of Ser36 by p66^Shc^ is required [7,10,11]. Therefore, an increased activation of p66^Shc^ might be enough to inhibit the Ras-MAPK pathway. However, the role of p66^Shc^ in transducing RTK signals is far from being completely understood.

There is vast literature showing that p66^Shc^ plays a major role in the response to oxidative and environmental stress stimuli [4,12,13,14,15,16,17,18] (Figure 1B). Kinases like c-Jun N-terminal kinase (JNK) or protein kinase C β (PKCβ), which are activated in response to stress stimuli, can phosphorylate a particular serine residue (Ser^36^) of p66^Shc^ within its CH2 domain [19,20]. This step is followed by a *cis-trans* isomerization by peptidyl-prolyl cis-trans isomerase 1 (Pin1), which allows the translocation of p66^Shc^ into the inter-membrane mitochondrial space, after it has been dephosphorylated by protein phosphatase 2A (PP2A). A more recent paper found that Ser^36^ might not be the crucial phosphorylation site to mediate the PKCβ response, while Ser^139^, Ser^213^, and Thr^206^ might be involved [21]. At the mitochondrial level, and without pro-apoptotic stimuli (such as H_2_O_2_ or UV radiation), p66^Shc^ is bound to high-molecular weight complexes and heat shock protein 70 (HSP70) or other proteins involved in the inter-membrane transport [22,23,24]. After stimulation, however, p66^Shc^ can interact with cytochrome c through its CB domain, generating reactive oxygen species (ROS), by diverting electrons from the mitochondrial electron transport chain (ETC) [4,15,18,25]. In this regard, it is worth mentioning that some authors, based on the structure of p66^Shc^, questioned its ability to be an acceptor of electrons from the ETC (reviewed in [5,26]). However, it should be noted that, in the absence of further experimental data to corroborate this notion, this remains a mere speculation. In any case, even if the exact mechanism might be still debated, it is well known that p66^Shc^ is involved in the production of ROS, and an excess in ROS production can interfere with many cellular processes and induce apoptosis. Apart from increasing mitochondrial ROS production, there are two other mechanisms whereby p66^Shc^ can increase ROS levels: (i) by decreasing the production of ROS scavengers through inhibition of forkhead box O (FOXO) transcription factors and (ii) by increasing the activity of membrane NADPH oxidase via Rac1 activation (reviewed in [5,15]). The involvement of p66^Shc^ in the induction of apoptosis is confirmed by the fact that its elimination or over-expression have opposite effects, making cells more resistant or more susceptible to apoptosis, respectively, ([12,16] and reviewed by [4,5,14,27]). However, the fact that p66^Shc^ favors ROS formation thereby stimulating apoptosis could be a too simplistic view, since both an anti-oxidant [28] and an anti-apoptotic behavior of p66^Shc^ [29] have been reported, albeit only in specific cell types and conditions. It was also reported that p66^Shc^ can participate in the induction of apoptosis, acting downstream of p53 [16]. The activation of p53 in response to H_2_O_2_ confers stability to the p66^Shc^ protein and probably an increase at the transcript level, since there is a p53-binding region within the *p66^Shc^* promoter [30]. Indeed, p53 can be activated even in the absence of p66^Shc^, but the cells become apoptosis-resistant in such conditions.

As discussed above, PKCβ can phosphorylate p66^Shc^, and a study pointed out a link between p66^Shc^ and the autophagic pathway [31]. Autophagy is a highly regulated process through which the cells can recycle components that are either unnecessary or malfunctioning. It is well known that starvation activates autophagy, and the authors demonstrated that p66^Shc^ can inhibit autophagy, following starvation in mouse embryonic fibroblasts (MEF) in a PKCβ-dependent manner. A recent paper investigated the induction of autophagy in vivo in the muscles of mice after downhill running, which is a type of exercise known to induce muscle damage, ROS production, and activation of the autophagic process [32]. Their data indicate that p66^Shc−/−^ mice have higher LC3 lipidation than wild type (WT) mice, but it is not further increased after exercise and other autophagic markers are not significantly different.

### 1.2. p66^Shc^ and Longevity

It was initially reported that deletion of the *p66^Shc^* gene was sufficient to cause an increase in the average and maximum longevity in mice [12]. Indeed, mice in which *p66^Shc^* was deleted had a 30% increase in their life-span compared with WT. These results were surprisingly similar to those obtained by putting mice under calorie restriction [33,34], but p66^Shc−/−^ mice were not leaner, nor did they eat less than WT. This observation supported the idea that a decreased ROS production was protective against the accumulation of DNA damages caused by free radicals, thereby delaying ageing and promoting an increase in life-span. Despite the inhibition of apoptosis, these mice did not show an increased susceptibility toward tumorigenesis. As already mentioned, p66^Shc−/−^ is a downstream mediator of p53 in the apoptotic pathway, but its deletion does not interfere with other p53-dependent pathways [16]. Indeed, p53^−/−^ mice displayed increased mortality due to spontaneous tumorigenesis, which was not observed in p66^Shc−/−^ mice. A more recent study on this matter dismantled the notion that p66^Shc^ regulates life-span: by using a higher number of mice compared with the original study, three different mouse strains (C57BL/6J, 129Sv, and a hybrid C57BL/6J-129Sv), and animals housed in two different facilities, p66^Shc−/−^ did not show increased life-span [35]. The authors noted how the average and maximum longevity of the WT mice were unusually low in the original study [12], and this could have been due to environmental stress. Moreover, the suspicion that p66^Shc^ was not involved in longevity determination was already raised by a study conducted on centenarian humans, in which it was found that the expression of p66^Shc^ in isolated fibroblasts was elevated, instead of being reduced [36].

Importantly, the role of p66^Shc^ in the determination of lifespan was further investigated in another study, wherein a telomerase RNA component (TERC^−/−^) and p66^Shc−/−^ double knockout mouse was generated. TERC^−/−^ mice have a decreased average lifespan, and it was observed that the concomitant deletion of p66^Shc^ was unable to restore this defect, while it was able to ameliorate other aspects, like sterility, weight loss, and multi-organ atrophy. To date, the exact phenotype of these mice has not been fully investigated [37]. In summary, it is possible that p66^Shc^ does not truly regulate life expectancy, but it is involved in the determination of health-span, which has a very strong translational impact to human pathology.

### 1.3. p66^Shc^, Body Weight Regulation, and Obesity

According to the authors of [12], the body weight of p66^Shc−/−^ mice was identical to that of WT mice, and so was food intake. In contrast, a more recent paper found that p66^Shc−/−^ mice were leaner, with body weight differences being mainly due to a decreased amount of abdominal and inguinal fat, particularly evident in males, while the weight of other organs was not different [38]. Our group also showed that, under standard diet, p66^Shc−/−^ were leaner than WT mice [39]. The observation that organ weight was not different between WT and knockout mice was confirmed by another study, in which no differences in total body weight or in fat-free mass was found, except at older ages (27-month-old animals) [40]. It was also shown that p66^Shc−/−^ mice subjected to a 5% calorie-restriction (CR) regimen, between 4 and 18-months-of-age, are leaner than WT mice, but not with a 40% CR [35]. The effect of calorie restriction on body weight was also studied in 18-month-old animals [41]: at baseline there were no differences between WT and knockout animals, and the same results were found when a 26% CR regimen for 2 months or a 40% CR regimen for 3 days was applied. The mechanisms whereby p66^Shc^ would regulate body weight are incompletely understood. On one side, there may be a heat dissipation from the ETC due to increased uncoupling in the adipose [38]. Furthermore, insulin is able to activate the production of H_2_O_2_ in pre-adipocytes from brown adipose tissue, but not if p66^Shc^ is ablated [38], and this event is necessary to modulate the activity of the Akt-Foxo1 pathway. In particular, if p66^Shc^ is missing, the phosphorylation of Akt is blunted. A proper response to insulin stimulation allows for the accumulation of triglycerides both in brown and white pre-adipocytes, by favoring their import and contemporaneously inhibiting β-oxidation processes.

Even if there is a general consensus about the fact that p66^Shc^ deletion confers protection against obesity, some conflicting data were found. It was shown by many authors that p66^Shc−/−^ mice are obesity-resistant, whether obesity is genetically- [39,42] or diet-induced [38,39,43]. However, in another study, a new p66^Shc−/−^ mouse model was generated, named ShcL [44], and some contrasting data were shown. ShcL mice were susceptible to, not protected from, diet-induced obesity, becoming more obese than WT animals in response to a high-fat diet (HFD). The authors showed that ShcL mice did not have perturbations in the expression pattern of p52^Shc^ and p46^Shc^, compared with the original p66^Shc−/−^ mice (called ShcP), while p46^Shc^ was increased in the adipose tissue of the ShcP; they reasoned that this might be the reason for the discrepancy in obesity. However, the idea that an increase in p46^Shc^ together with the absence of p66^Shc^ in the adipocytes, eventually coupled with a decreased expression of p52^Shc^, is responsible for a decreased fat accumulation was not supported by further experimental data. In addition, we did not find any increase in adipose tissue p46^Shc^ protein expression in ShcP mice [39].

Very few studies have focused on adipokines. In particular, it was demonstrated that in lean p66^Shc−/−^ mice there were decreased plasma levels of leptin and adiponectin [39,45]. The same was also demonstrated in obese knockout animals for adiponectin [39] and leptin [45]. One study, in contrast, found no differences in plasma leptin concentration between WT and p66^Shc−/−^ mice, but only increased plasma leptin levels in females compared with males, regardless of genotype, when animals were prenatally exposed to HFD [46]. Regarding adiponectin, it was higher in knockout females compared with WT, and also in females versus males, but only in p66^Shc−/−^. Adiponectin was also measured in primary brown adipocytes [45] or in the adipose tissue [46], finding a decrease in p66^Shc−/−^ adipocytes or an increase only in p66^Shc−/−^ females, respectively. The concentration of circulating plasminogen activator inhibitor 1 (PAI-1) was similar between WT and knockout animals, which was increased in obese Lep^ob/ob^ animals, regardless of genotype [39], while its expression in white adipose tissue, measured by quantitative PCR, was decreased [45]. Finally, TNFα production was decreased also in p66^Shc−/−^ mice, both in the plasma of obese animals and in primary brown adipocytes [45].

### 1.4. p66^Shc^, Diabetes, and the IGF-1 Axis

The possibility that p66^Shc^ regulates body weight and that its deletion improves obesity, made p66^Shc^ a possible candidate gene against obesity-related diseases. [47]

Furthermore, based on the role of p66^Shc^ in ROS production, there is a consensus about the ability of p66^Shc−/−^ mice to counteract many side-effects of pathologies commonly attributed to oxidative stress, including chronic diabetic complications. In fact, it has been demonstrated that the absence of p66^Shc^ confers protection toward diabetes-induced endothelial damage [48,49], diabetic nephropathy [46], and diabetic cardiomyopathy [50] and improves the healing of diabetic ulcers [51]. The exact mechanisms at work have never been clearly dissected, and there may be several pathways affected by p66^Shc^ deletion in addition to the regulation of cellular oxidant status.

It was reported that the insulin-like growth factor 1 receptor (IGF1-receptor) can phosphorylate p66^Shc^, and in MEFs derived from mice where the expression of IGF1-receptor was reduced (IGF-1R^+/−^ mice) there was also a reduced tyrosine phosphorylation of p66^Shc^ and p52^Shc^ [52]. IGF1 stimulation is also able to induce the phosphorylation of p66^Shc^ in tyrosine residues in L6 myoblasts [53], and the silencing of p66^Shc^ leads to abnormal phosphorylation of extracellular signal-regulated kinase 1/2 (ERK1/2), which is elevated in basal conditions and blunted after IGF1 stimulation. Moreover, the reduced expression of p66^Shc^ caused an increased glucose uptake in basal conditions, preeminently due to an ERK-mediated remodeling of the actin cytoskeleton, but also to an increase of GLUT1 and GLUT3, both at the protein and mRNA levels [54].

### 1.5. Role of p66^Shc^ in the Regulation of Glucose Homeostasis

Many studies tried to shed light on the role of p66^Shc^ in insulin and glucose metabolism. This is reasonable, as Shc adapter proteins can interact and transduce the signaling evoked by insulin receptors (reviewed in [55]). Moreover, it was demonstrated that IRS-1, p66^Shc^, and S6K can associate to form a complex (reviewed in [56]). However, as already noted for body weight and obesity, contrasting data can be found in the literature in regard to the role of p66^Shc^ in glucose and insulin tolerance at the whole-body level. As reported above, GLUT1 and GLUT3 were upregulated in L6 myoblasts with reduced p66^Shc^ expression. Moreover, basal glucose transport was increased, while the adenoviral-mediated overexpression of p66^Shc^ produced opposite effects [54]. Using different cell types (HeLa and MEFs), it was confirmed that p66^Shc^ deficiency enhances glucose uptake [57]. Also, cells have an increased proportion of metabolites for fatty acids biosynthesis. Another partial confirmation came from a study conducted in the skeletal muscles of p66^Shc−/−^ mice, wherein glucose uptake was not studied in detail, but it was found that glucose content was similar to that of fed WT animals, and glycogen was more abundant in knockout muscles, either in fed or starved conditions [58], indicating that glucose uptake in knockout animals was probably not impaired. In primary adipocytes, basal glucose uptake was found to be identical between WT and p66^Shc−/−^ but increased after insulin stimulation only in knockout cells [42]. Using the aforementioned ShcL p66^Shc−/−^ knockout mice, it was confirmed that insulin-stimulated glucose uptake was increased in cultured adipocytes, compared with that of WT-derived adipocytes [44]. However, this matter may be more complicated than it seems. In the same paper [44], using the initial p66^Shc−/−^ mice (ShcP) as in [42], opposing results were found regarding insulin-stimulated glucose uptake in adipocytes, which was decreased in the first and increased in the latter, respectively. An attempt to reconcile these contrasting data was made in a more recent study [56], where the authors discussed the possibility that p66^Shc^ plays both a positive and a negative role on the insulin pathway, by acting upstream and downstream of mTOR/S6K. Basically, it was proposed that in WT obese mice there is insulin desensitization in the adipose tissue due to constitutive activation of S6K (and its substrate S6), which leads to IRS-1 degradation. In parallel, decreased PI3K recruitment to IRS-1 impairs downstream Akt signaling. Finally, we also reported that glucose uptake in isolated skeletal muscles was lower in p66^Shc−/−^ mice, compared that with WT, after insulin stimulation [39].

Some studies reported an increased lactate production in response to p66^Shc^ deletion. This was demonstrated in immortalized p66^Shc−/−^ MEFs as a result of increased anaerobic glycolysis and decreased mitochondrial respiration [59], and confirmed in the same cell type and also in HeLa cells [57]. In the latter paper, the authors showed that p66^Shc^ deficiency in HeLa cells enhances the glycolytic metabolism, favored by the concomitant activation of the pentose phosphate and hexosamine pathways, which contributes to the maintenance of a proper redox balance within the cell (by provision of NAPH) and provides a positive feedback on the signaling [57]. On the other hand, when p66^Shc^ expression was restored in p66^Shc−/−^ MEF cells, glycolytic metabolism was impaired. Interestingly, this paper identified a link between p66^Shc^ and mTOR signaling and it was shown that activation of mTOR is associated with an increased anabolic metabolism and protein synthesis. In particular, both S6K and Akt, targets of mTORC1 and mTORC2, respectively, were phosphorylated in p66^Shc−/−^ compared with control HeLa cells after serum stimulation, but not if cells were pre-incubated with the mTOR inhibitor Torin. Consistently, an increased production of lactate and citrate was also observed in the skeletal muscles of p66^Shc−/−^ mice [58], but in this case the authors showed a decreased glycolytic capacity, both in a fed and fasted state, as suggested by a decreased activity of key glycolytic enzymes, such as hexokinase, phosphofructokinase, and pyruvate kinase.

The relationship between p66^Shc^ and glucose homeostasis was also studied in obese mice, and conflicting results have been obtained even in this case. p66^Shc−/−^Lep^Ob/Ob^ double knockout mice on a mixed genetic background (C57Bl/6J and 129Sv) had improved insulin sensitivity (similar to that of WT lean mice) and glucose tolerance compared with p66Shc^WT^ Lep^Ob/Ob^ animals, but were still glucose intolerant [42]. No differences in fasting glucose between lean WT and p66^Shc−/−^ mice were reported, nor in glucose tolerance tests (GTT) and insulin tolerance tests (ITT). Another study, however, found that lean p66^Shc−/−^ were more insulin sensitive and glucose tolerant than WT animals [44] at 3 months of age. At 24 months, only insulin sensitivity was improved, in comparison with WT animals. Moreover, insulin sensitivity was reported to be higher in muscle samples from both ShcL and ShcP mice than from control WT. The same paper reported an improved insulin sensitivity in ShcP mice even after a high fat diet (HFD), which was lost in ShcL mice, at least in the liver. However, our data depicted a different scenario in mice on a pure C57Bl/6J background: worsened glucose tolerance in 18-week-old and more insulin resistance in 30-week-old p66^Shc−/−^ lean compared with WT lean animals, while obese p66^Shc−/−^ were more insulin resistant and equally glucose intolerant [39]. We further confirmed that obese p66^Shc−/−^ are not protected from insulin resistance and glucose intolerance [43]. These data on mice were supported by data on human samples, in which a decreased expression of *p66^Shc^* in the visceral adipose tissue was associated with a lower BMI, but without any improvement in diabetes, dyslipidemia, or hypertension [39]. We and others found that the muscles of obese p66^Shc−/−^ mice have increased ectopic fat accumulation, which in part can explain these results [32,39,44]. Moreover, we also demonstrated that the microbiota hosted by p66^Shc−/−^ mice is different from that of WT controls, and that this might explain the differences between studies [43]. Finally, a recent paper studied the metabolic effect on the offspring of WT and p66^Shc−/−^ where dams were given a HFD before and during pregnancy. In this experiment, p66^Shc−/−^ progeny was protected from the deleterious effect of the diet, and that was especially evident for females [46]. However, p66^Shc−/−^ mice were more insulin resistant than WT animals (9 weeks after birth), and this was worsened in animals exposed to pre-natal HFD. WT and knockout males showed a similar response to glucose in GTT, while p66^Shc−/−^ females had an improved glucose tolerance, especially evident in the HFD group.

### 1.6. Body Temperature Regulation, Respiration, and Energy Expenditure

Interestingly, some studies indicated a possible link between p66^Shc^ and body temperature regulation. In the paper of Berniakovich and colleagues [38], it was shown that p66^Shc−/−^ mice had a higher basal body temperature (at 22 °C external temperature), compared with WT animals. This might be due to an increased metabolic activity in the brown adipose tissue (BAT), since p66^Shc−/−^ express higher amounts of uncoupling protein 1 (UCP1) in this tissue [38]. In the same work, it was demonstrated that the deletion of p66^Shc^ had a dramatic impact on cold adaptation. When housed at 5 °C, the body temperature of these mice dropped by about 6 °C, compared with a halved change in WT animals. The trough was reached more rapidly in knockout than in WT animals: after 3 or 4 h, respectively [38]. However, body temperature returned to normal values within 6 h, both in WT and p66^Shc−/−^ mice, so even if cold adaptation was affected, thermogenesis was not impaired. A possible explanation to this phenomenon comes from a lower thermal insulation in the knockout mice, due to a decreased white fat mass (as previously discussed). A more recent study confirmed this notion by demonstrating a negative selection toward p66^Shc−/−^ mice when left in large outdoor enclosures for one year [45]. This study is particularly relevant, as it sheds light on the reason why p66^Shc^ was phylogenetically conserved, despite its role in the induction of the oxidative stress. p66^Shc^ might be important to promote survival in conditions of environmental stress, whilst its metabolic role might be competitively disadvantageous or detrimental in modern life-style conditions, favoring the development of obesity and metabolic syndrome. In this regard, p66^Shc^ may be considered a typical thrifty gene [60].

Concerning respiration and energy expenditure, p66^Shc−/−^ male mice have a higher oxygen consumption at basal level, and a slightly higher energy expenditure than wild-type animals [38]. A more recent study found rather different results, as the respiratory quotient (calculated as the ratio between the volume of CO_2_ produced and the volume of the O_2_ consumed) was increased in p66^Shc−/−^ mice compared with WT animals, which is consistent with an increased glucose utilization in knockout mice [40]. In this study, energy expenditure was also measured, and p66^Shc−/−^ mice displayed lower values than WT mice. However, when fat-free mass was taken into account instead of total body weight, this difference was no more statistically significant. The response of p66^Shc−/−^ mice to calorie restriction was also investigated in 18-month-old animals [41]. When a 26% CR was applied for 2 months there were no differences between WT and knockout mice. However, after 24 h during the 3 days of 40% CR there was a significantly lower energy expenditure during the light phase, compared with the WT animals, but in this case, fat-free mass was not measured.

## 2. Conclusions and Perspectives

The great majority of the in vivo studies on p66^Shc^ were conducted on one single p66^Shc−/−^ mouse strain [12]. This mouse model was originally generated in the 129Sv strain, and it was afterwards backcrossed to the C57BL/6J strain, and then crossed with other mouse models, such as p53^−/−^, TERC^−/−^, and Lep^Ob/Ob^ [16,35,37,39,42]. The availability of this knockout was surely fundamental in the exploration of the metabolic role of p66^Shc^ and in confirming results that were produced in cell lines, wherein p66^Shc^ was silenced or deleted. However, relying on a single mouse model can be a limitation, and independent confirmations are required. Another p66^Shc−/−^, named ShcL, was made available recently, and some results were surprisingly in contrast with previous data [44]. To date, this mouse was used only in the referred paper. More importantly, it will be extremely helpful to develop tissue-specific and inducible p66^Shc^-knockout mice to better dissect the role of p66^Shc^ in different tissues and at different developmental stages or before/after environmental stresses (e.g., HFD). As shown in the present review, looking at different ages with different duration of HFD regimens, starting the diet at different ages can lead to contrasting results, which sometimes appear difficult to reconcile (Table 1). As a final complication, we showed that p66^Shc^ depletion can also influence gut microbiota, which in turns affects the metabolism of the mouse. Altogether, these notions encourage exploring further the role of p66^Shc^ in the regulation of body weight and metabolism. In view of the obesity and diabetes epidemic, p66^Shc^ may represent a promising therapeutic target with enormous implications for human health.

## Figures and Tables

**Table 1:** Summary of the contrasting results obtained on the role of p66^Shc^ in physiological conditions and metabolic diseases. WT = wild type.

Condition	In Favor	Against
Increased longevity	Deletion of p66^Shc^ increases average and maximum longevity [12]	No increase in lifespan of p66^Shc−/−^ mice [35] High expression of p66^Shc^ in centenarians [36] Deletion of p66Shc does not rescue TREC deficiency [37]
Bodyweight regulation	p66^Shc−/−^ mice are leaner than age-matched WT [35,38,39,40,43]	p66^Shc^ deletion has no influence on bodyweight [12,40,41]
Protection from obesity	Knockout mice gain less weight than WT controls [38,39,42,43]	Knockout mice gain more weight than WT controls [44]
Improved glucose homeostasis	Glucose uptake is increased in the absence of p66^Shc^ [42,44,54,57,58] Increased glycolysis [57,59] Improved glucose tolerance and/or insulin sensitivity in lean [44] and obese knockout animals [42]	Glucose uptake is not increased [42] or even decreased [39,44] in the absence of p66^Shc^ Decreased glycolysis [58] Similar or worsened glucose tolerance and/or insulin sensitivity in lean [39,43,44] and obese knockout animals [39,43,44]
